# Measurement of gluconeogenesis by ^2^H_2_O labeling and mass isotopomer distribution analysis

**DOI:** 10.1016/j.jbc.2023.105206

**Published:** 2023-09-01

**Authors:** Naveed Ziari, Marc Hellerstein

**Affiliations:** Department of Nutritional Sciences & Toxicology, University of California, Berkeley, California, USA

**Keywords:** mass spectrometry, glucose homeostasis, endogeneous glucose production, gluconeogenesis, deuterium labeling, diabetes

## Abstract

The gluconeogenesis pathway, which converts nonsugar molecules into glucose, is critical for maintaining glucose homeostasis. Techniques that measure flux through this pathway are invaluable for studying metabolic diseases such as diabetes that are associated with dysregulation of this pathway. We introduce a new method that measures fractional gluconeogenesis by heavy water labeling and gas chromatographic-mass spectrometric analysis. This technique circumvents cumbersome benchwork or inference of positionality from mass spectra. The enrichment and pattern of deuterium label on glucose is quantified by use of mass isotopomer distribution analysis, which informs on how much of glucose-6-phosphate-derived glucose comes from the gluconeogenesis (GNG) pathway. We use an *in vivo* model of the GNG pathway that is based on previously published models but offers a new approach to calculating GNG pathway and subpathway contributions using combinatorial probabilities. We demonstrated that this method accurately quantifies fractional GNG through experiments that perturb flux through the pathway and by probing analytical sensitivity. While this method was developed in mice, the results suggest that it is translatable to humans in a clinical setting.

Glucose homeostasis is critical to metabolic health, and its regulation depends heavily on the gluconeogenesis (GNG) pathway, which synthesizes glucose from nonsugar substrates, primarily in the liver (1). Dysregulation of this pathway is implicated heavily in metabolic diseases such as diabetes (2). The ability to measure flux through this pathway is therefore of vital importance to metabolism and pharmaceutical research, and more specifically, to measure liver activity and energy balance.

Measurement of GNG has proved itself to be both tricky and oftentimes elusive (3, 4). Static measurements such as mRNA expression or enzymology infer pathway activity indirectly. For instance, the gateway enzyme and an important control point of this pathway, phosphoenolpyruvate carboxykinase (PEPCK), has little to no correlation with GNG flux (5). The discrepancy or lack of concordance between mRNA or protein levels and flux through a pathway is not unusual (6). Measurement of GNG, or more generally of any flux in cellular metabolism, requires isotopic labeling and tracing. There currently exist a number of isotopic methods to measure this pathway; however, uncertainty and controversy remain in the field at large as to which method is most accurate and implementable (3, 7). We present here a method that measures fractional gluconeogenesis by deuterium labeling and mass spectrometry using mass isotopomer distribution analysis (MIDA).

In broad terms, the point of investigation is into the source of blood sugar in the assay of hepatic glucose production. Endogenous glucose production (EGP) mostly occurs in the liver, where it could be either from glycogen breakdown or GNG, and GNG itself has different arms and subpathways (Fig. 1). MIDA allows calculation of fractional gluconeogenesis (GNG), which combined with fractional glycogenolysis (GGL), sum to unity for EGP. Phrased differently, the method calculates the ratio of glycogen breakdown *versus* GNG fluxes, providing a snapshot of hepatic glucose production. The measurement of absolute GNG, which means the total amount of glucose being produced through the GNG pathway, is possible with the use of a second tracer to determine the rate of appearance (Ra) of glucose (8, 9). Glucose exported from the liver is also diluted by dietary glucose in the fed state.

**Figure 1:**
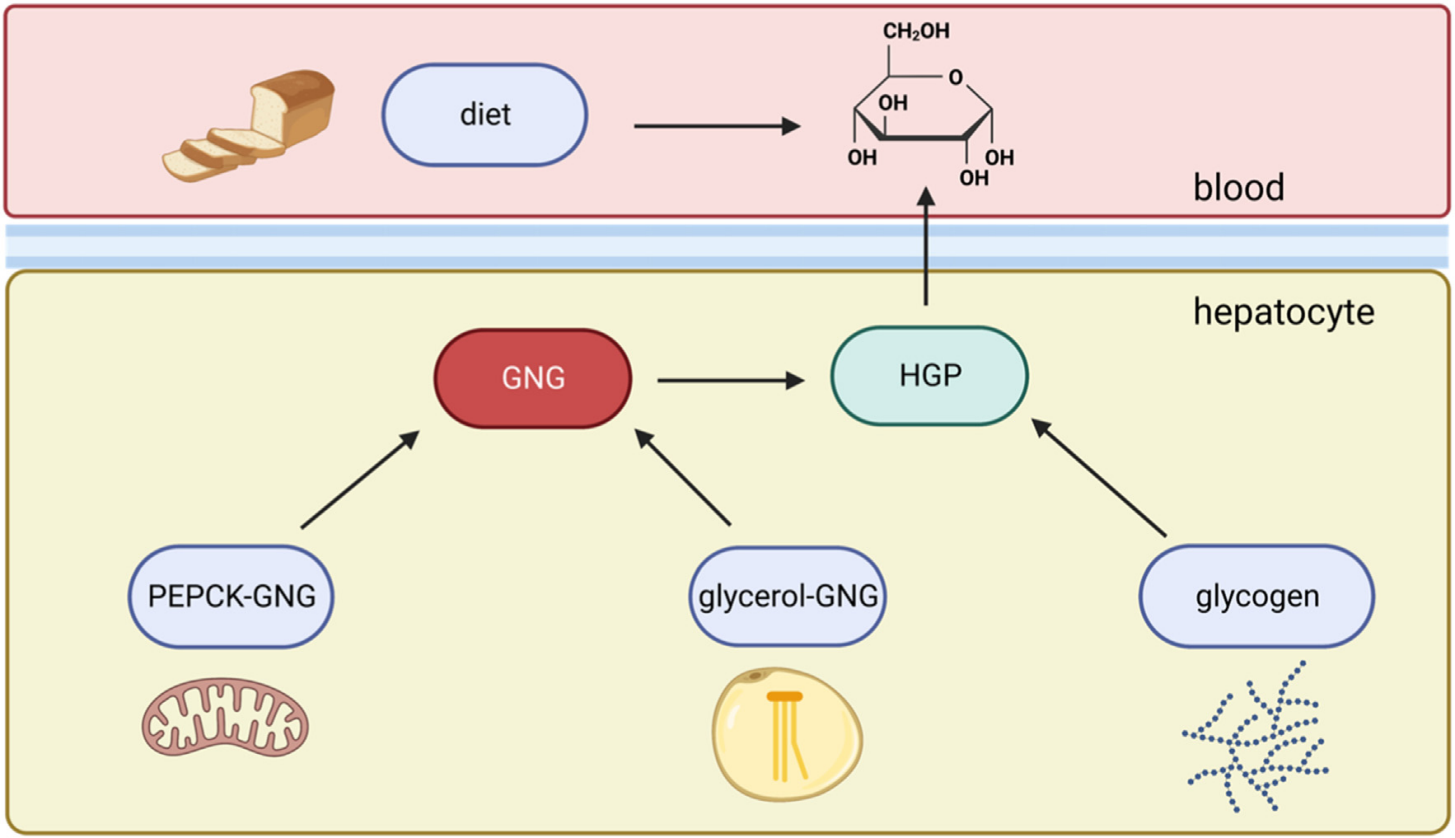
**Schematic of pathway contributions to hepatic glucose production.** Total GNG consists of PEPCK-GNG and glycerol-GNG, which meet at the triose phosphates. Glycogenolysis (GGL) meets at glucose-6-phosphate, which gets converted to glucose by glucose-6-phosphatase and exported out of the cell into circulation. GNG, gluconeogenesis; PEPCK, phosphoenolpyruvate carboxykinase.

There are a number of methods that use heavy water to measure GNG, all of which are premised on the fact that each GNG subpathway has a unique set of enzymes reacting and exchanging nonacidic protons with solvent water (10, 11). Depending on which pathway a glucose molecule traversed, it incurs a differing amount and distribution of isotopically labeled hydrogen atoms in the presence of heavy water (deuterium oxide, ^2^H_2_O) (11). By measuring the amount and pattern of label on glucose using MIDA and GC-MS, we show here that it is possible to accurately determine fractional GNG. This method is unique in that it does not try to account for or resolve label positionality on the glucose molecule (positional isotopomers), but rather calculates the total amount and statistical pattern of label on glucose (mass isotopomers) using the MIDA algorithm, which can inform on how much comes from GNG pathways. This approach also represents a potential paradigm for global metabolomic flux measurements if extended to other intermediary metabolites.

## Results

### Glycerol contribution to GNG in the fasted state

**Figure 2:**
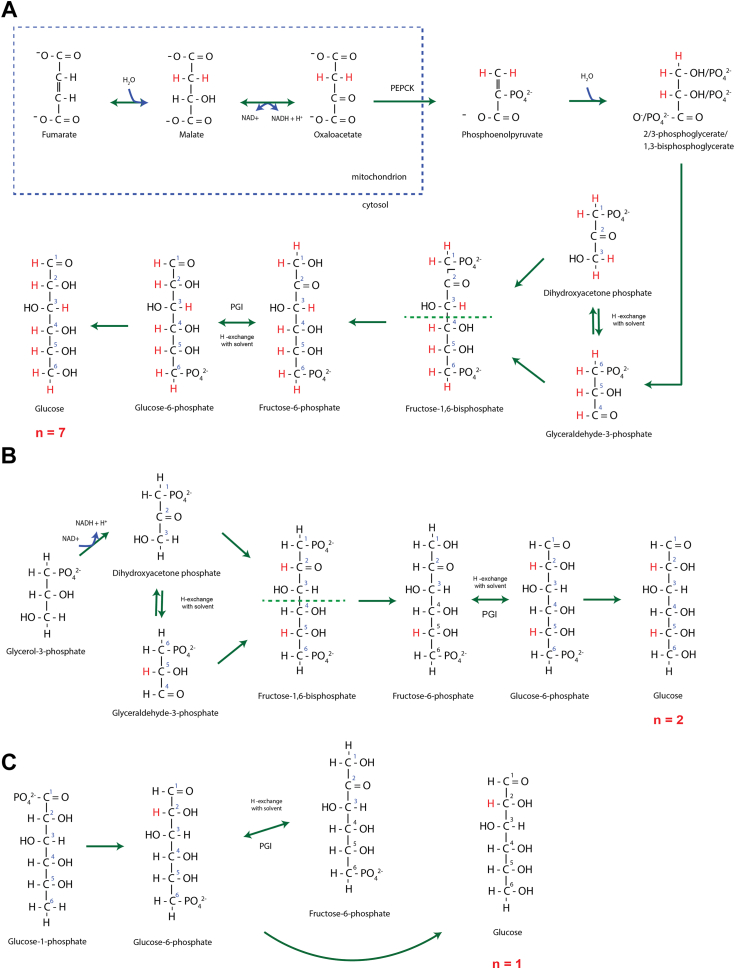
**Illustration of H-exchange in C-H bonds of glucose during enzymatic reactions and resulting*****n.*** For (*A*) PEPCK-GNG: all nonacidic hydrogens become labeled (n = 7); (*B*) glycerol-GNG: carbon-5 is labeled by triose-phosphate isomerase and then carbon-2 at phosphoglucose-isomerase (PGI, n = 2); (*C*) glycogenolysis (GGL) only encounters PGI and obtains only one label at carbon-2 (n = 1). GNG, gluconeogenesis; PEPCK, phosphoenolpyruvate carboxykinase; PGI, phosphoglucose isomerase.

Total GNG can be divided into glycerol-GNG and PEPCK-GNG (Fig. 1), where each pathway has a different number of exchangeable hydrogen positions (*n*). For glycerol-GNG, n = 2 while for PEPCK-GNG, n = 7 (Fig. 2). In a fasted state in rodents there is essentially no contribution from liver glycogen, which makes figuring out how much comes from glycerol *versus* PEPCK straightforward. Contributions from the two pathways add up to 100% and each pathway has a unique labeling pattern that reflects relative isotope enrichments in single- and double-labeled nominal masses of glucose (EM2/EM1) at any given body ^2^H_2_O enrichment (*p*). Combinatorial probabilities teach that n = 2 gives different EM2/EM1 than n = 7. The experimentally derived EM2/EM1 therefore informs on how much plasma glucose comes from each subpathway of GNG (see Experimental procedures—MIDA calculations). More precisely, in a fasted state, when f(GNG) is >95%, or<5% contribution from liver glycogen breakdown, this affords the luxury of determining with heavy water labeling the contribution of glycerol relative to PEPCK to the triose-phosphate pool, which is the more proximal or upstream precursor pool of GNG in the liver.

**Figure 3:**
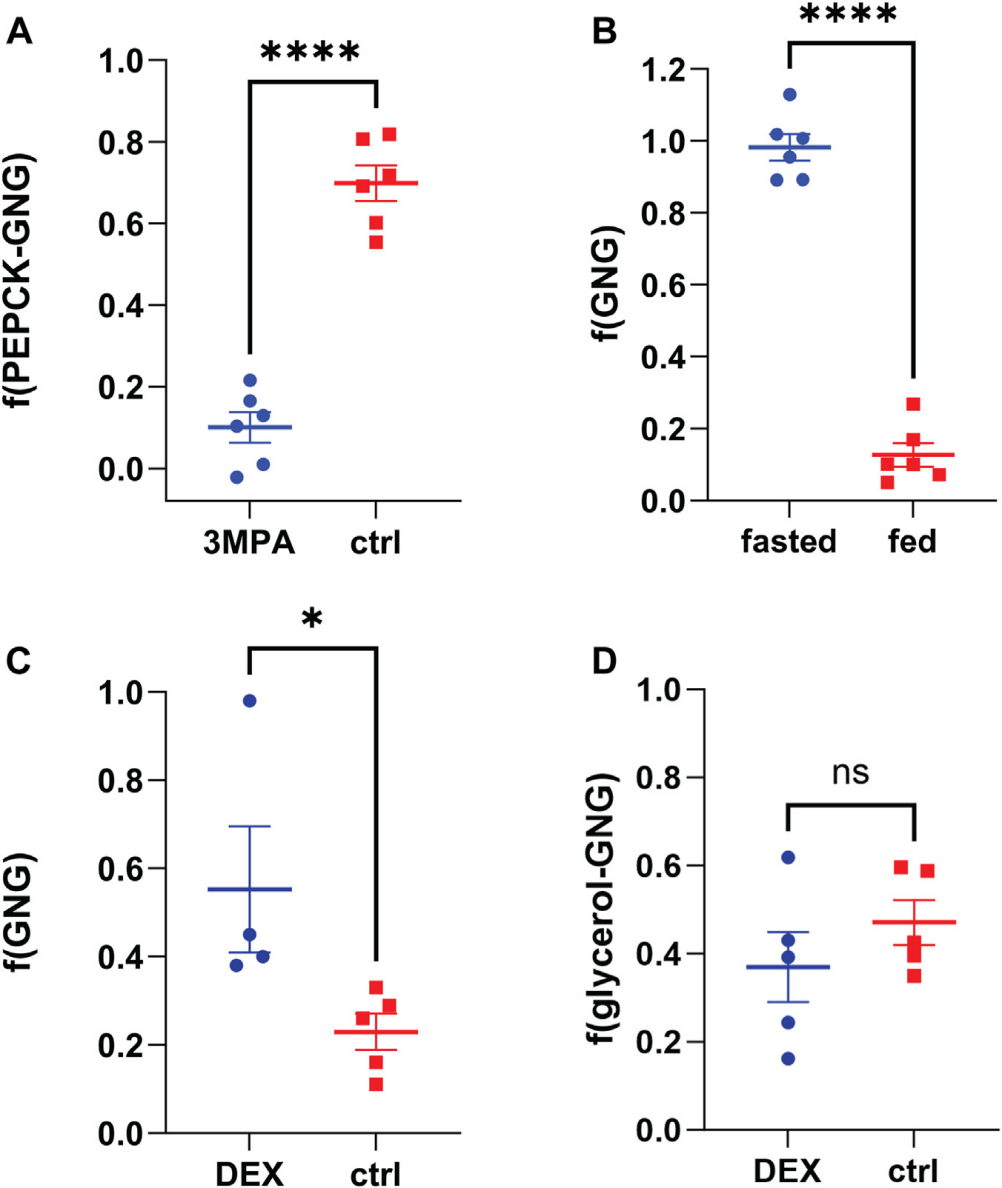
**Results with heavy water labeling for plasma glucose metabolic sources in mice.***A*, in fasted mice in the control condition, contribution of PEPCK to total GNG is around 70% treatment with 3-MPA significantly downregulated flux through PEPCK GNG relative to glycerol-GNG; (*B*) in 24-h fasted mice, plasma glucose was almost completely from GNG whereas in *ad libitum* fed mice, plasma glucose was mostly from glycogen. *C*, dexamethasone treatment in *ad libitum* fed mice increases f(GNG) on *ad-libitum* fed mice; *D*, lack of effect of dexamethasone treatment on glycerol contribution to triose-phosphate pool and f(GNG) in fasted mice, where f(glycerol-GNG) = 1 – f(PEPCK-GNG). GNG, gluconeogenesis; MPA, mercaptopicolinic acid; PEPCK, phosphoenolpyruvate carboxykinase.

Around one-third of the triose-phosphate pool is calculated to come from glycerol based on heavy water labeling in the fasted state (Fig. 3, *A* and *F*(PEPCK-GNG) = 0.70 ± 0.04), which is in agreement with other studies in mice and in humans (9, 12). In addition, we found that administration of 3-mercaptopicolinic acid (3-MPA), a known inhibitor of PEPCK (13), significantly increases the proportion of flux through glycerol GNG relative to PEPCK such that the EM2/EM-calculated f(PEPCK-GNG) = 0.10 ± 0.037 (statistically highly significant, with *p* < 0.0001, Fig. 3*A*).

### Glycerol contribution to GNG in fed state

In a fed state, there is also contribution from liver glycogen mobilization, so it is not possible to deconvolve contributions of PEPCK-GNG *versus* glycerol-GNG, when there are two or more unknowns that are conceptualized as degrees of freedom (PEPCK-GNG, glycerol-GNG, and glycogen). It is necessary to know the glycerol contribution in the fed state to calculate f(GNG) *versus* f(GGL) contributions in the fed state. Toward that end, administration of [2-^13^C]-glycerol was used to calculate glycerol contribution to the triose-phosphate pool and overall contribution to EGP.

Glucose is a condensation product formed by two triose subunits and can be conceptualized as a polymer. A combinatorial process with n = 2 is therefore amenable to using MIDA, not only to calculate f(GNG), but also to calculate the contribution of plasma glycerol to the triose-P pool. Previous articles from our lab have described this method in detail (14, 15). In brief, the contribution of plasma glycerol to the triose-phosphate pool is calculated by comparing the enrichment of plasma glycerol to that of p (the calculated triose-phosphate precursor pool enrichment for GNG), the latter being determined by MIDA based on combinatorial probabilities. Multiplying the contribution for free glycerol to triose-phospate by the total contribution to glucose from GNG [f(GNG)] reveals how much of EGP comes from glycerol *versus* PEPCK in a fed state.

**Figure 4:**
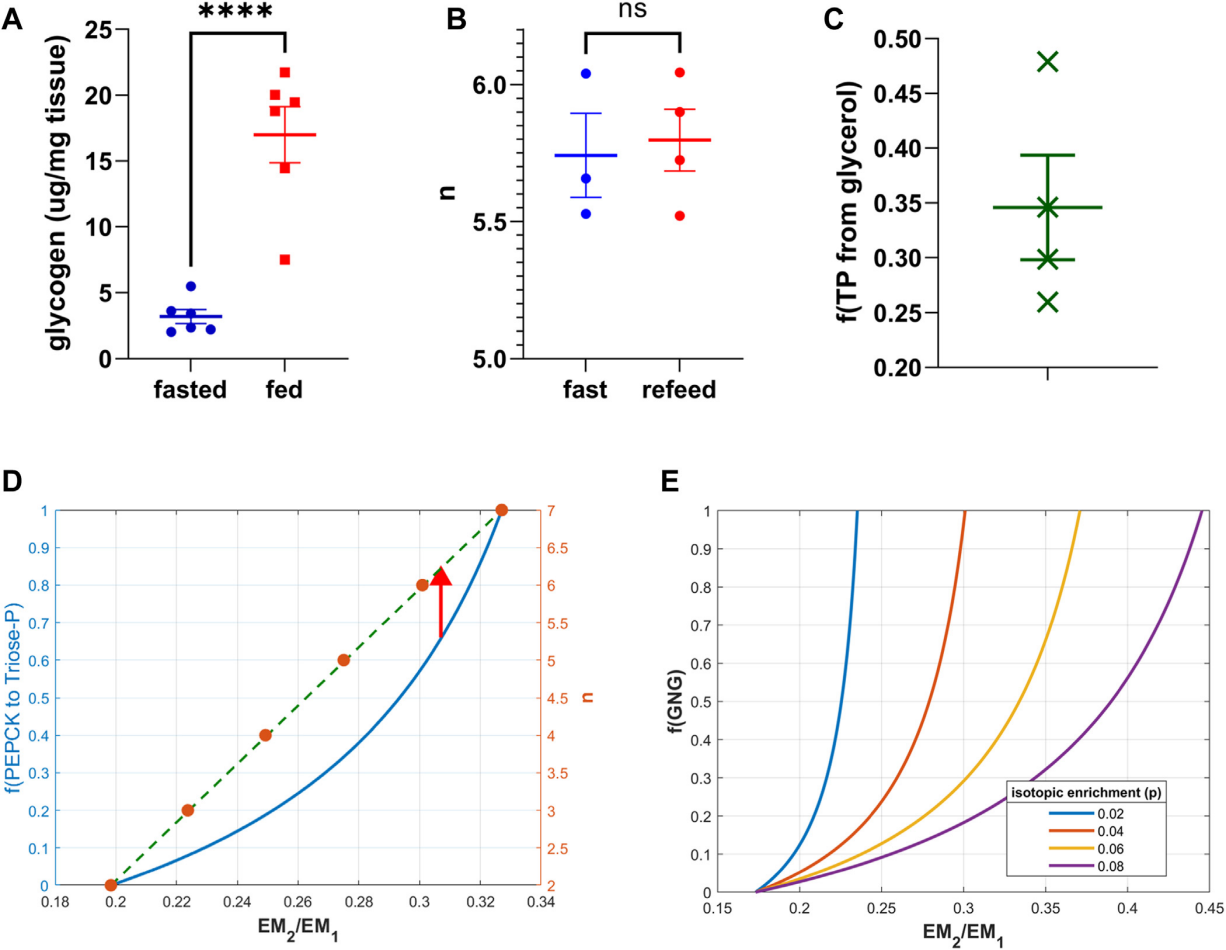
**Validation experiments and features of the mixture model.***A*, glycogen measurements for the experiment are shown in Figure 3*B*. Glycogen concentrations in the liver are markedly reduced in the fasted state; (*B*) the lack of effect on calculated n in the brief refeeding experiment; (*C*) infusion of [2-^13^C]-glycerol allows direct calculation of glycerol contribution to the triose phosphate pool in the ad-libitum fed state; (*D*) calculation of *n* for total GNG from heavy water labeling. *Left y*-axis and *blue curve* are mixture of PEPCK-GNG (n = 7) and glycerol-GNG (n = 2), *right y*-axis and *orange dots* represent *n* values that would generate EM2/EM1 ratio if these were a homogenous population of glucose molecules from a single GNG pathway. Positioning 0.66 for f(PEPCK to triose-phosphate) to denote one-third contribution from glycerol (*vertical red arrow*) has the same EM2/EM1 ratio compatible with a homogenous population of *n* = 6; *E*, f(GNG) as a function of EM2/EM1 at various body water enrichments *p*. GNG, gluconeogenesis; PEPCK, phosphoenolpyruvate carboxykinase.

We find that the contribution of glycerol to GNG during the fed state is similar to that of the fasted state, around one-third (0.345 ± 0.095, Fig. 4*C*), consistent with previous studies that also yield similar results (9, 12, 16).

There is more variation in the fed state among replicates (SEM = 0.0336), primarily because of fluctuations in f(GNG). Ultimately, the contribution of plasma glycerol to blood glucose is rather small being around 5 to 10%, as described in previous studies (9).

### Calculation of f(GNG) by ^2^H_2_O labeling

Based on the results shown here (Figs. 3 and 4*C*) and in other studies (12, 17, 18), we define total GNG from ^2^H_2_O labeling by simulating a mixture of two-thirds PEPCK (n = 7) and one-third glycerol GNG (n = 2). This labeling pattern (*i.e.* EM2/EM1, or R) at any *p* is compatible with a mixed glucose population with calculated n = 6. Mixing populations of relatively disparate *n* values (*i.e.* pathways that encounter significantly different number of enzymatic reactions that exchange with solvent water), is curvilinear and the ratio of pathway contributions is determined by referencing the generated curve. The exact value of *n* that generates this two-thirds and one-thirds of these two subpathway inputs is 6.12 (Fig. 4*D*), but an integer approximation is more straightforward to generate MIDA tables and to ultimately calculate f(GNG). No matter what the actual contribution of glycerol is to total GNG, which could vary, we demonstrate below (see “Variation of glycerol contribution to triose-P and effect on f(GNG)”) that designating n for total GNG as six accurately quantifies f(GNG) within feasible ranges of glycerol contribution to triose-P.

Accordingly, the calculation of fractional GNG in the fed state involves mixing a population of n = 6 (total GNG) and n = 1 (glycogen), which sum to unity (100% contribution to glucose) using the mixture model described in Experimental procedures—MIDA calculations. We reference Table 1 generated by this method, where the experimentally derived EM2/EM1 ratio quantifies f(GNG) parameterized by the body water enrichment *p*.

**Table 1:** Tabulation of EM2/EM1 values over gradient of f(GNG) and body water enrichment *p*

f(GNG) (%)→p(%)↓	0	10	20	30	40	50	60	70	80	90	100
0.5	0.173155	0.179123	0.182154	0.183988	0.185218	0.186099	0.186762	0.187279	0.187693	0.188032	0.188315
1	0.173155	0.184984	0.191088	0.194813	0.197324	0.19913	0.200492	0.201556	0.20241	0.203111	0.203696
1.5	0.173155	0.190738	0.199953	0.205626	0.20947	0.212246	0.214346	0.215989	0.21731	0.218395	0.219303
2	0.173155	0.196383	0.208747	0.216424	0.221654	0.225447	0.228323	0.230579	0.232395	0.23389	0.235141
2.5	0.173155	0.20192	0.217466	0.227202	0.233873	0.238729	0.242423	0.245326	0.247669	0.249599	0.251216
3	0.173155	0.207346	0.226106	0.237958	0.246125	0.252093	0.256646	0.260233	0.263133	0.265525	0.267532
3.5	0.173155	0.212663	0.234666	0.248689	0.258406	0.265537	0.270993	0.275301	0.278791	0.281674	0.284096
4	0.173155	0.217869	0.243143	0.25939	0.270714	0.279059	0.285462	0.290532	0.294645	0.298049	0.300913
4.5	0.173155	0.222964	0.251533	0.270059	0.283047	0.292657	0.300055	0.305926	0.310699	0.314655	0.317988
5	0.173155	0.227949	0.259834	0.280692	0.295401	0.30633	0.314771	0.321486	0.326956	0.331497	0.335328
5.5	0.173155	0.232823	0.268043	0.291286	0.307773	0.320076	0.329609	0.337212	0.343418	0.348579	0.352939
6	0.173155	0.237588	0.276159	0.301838	0.320161	0.333894	0.34457	0.353107	0.360089	0.365906	0.370827
6.5	0.173155	0.242242	0.28418	0.312344	0.332562	0.347782	0.359653	0.369171	0.376972	0.383483	0.388999
7	0.173155	0.246787	0.292102	0.322801	0.344973	0.361738	0.374858	0.385406	0.394071	0.401315	0.407461
7.5	0.173155	0.251223	0.299925	0.333206	0.357391	0.37576	0.390185	0.401814	0.411388	0.419407	0.426222
8	0.173155	0.255551	0.307646	0.343557	0.369813	0.389846	0.405633	0.418396	0.428927	0.437765	0.445287

Locating the experimentally derived EM2/EM1 value at the measured *p* yields f(GNG). Using the arithmetic mean is accurate in the small windows between the 0.5% intervals for p shown in the table.

### Prolonged fasting increases f(GNG)

In order to validate this model, we fasted mice for 24 h and found that f(GNG) approaches 100%, while in the *ad libitum* fed state, f(GNG) had < 15% contribution (0.983 ± 0.037 *versus* 0.127 ± 0.033, *p* < 0.0001) (Fig. 3*B*). Glycogen measurements show a profound reduction in liver glycogen content in the fasted mice (3.2 ± 0.53 *versus* 17.0 ± 2.1 μg/mg tissue, *p* < 0.0001) (Fig. 4*A*). Any glycogen present in the fasted state, as shown by these results and the lack of dietary glucose, might itself have come from the GNG pathway, and therefore still represent net GNG and reflect that labeling pattern. Previous studies have also investigated in greater detail the source of glycogen in a fasted state (19). Studies done in humans find that f(GNG) increases proportionally with the duration of fasting to asymptotically approach 95 to 100% (10), consistent with the method reported here.

### Glucocorticoid treatment increases f(GNG) but not the relative proportions from glycerol *versus* PEPCK

Glucocorticoids upregulate GNG (20, 21). We administered dexamethasone for 8 days to female 129S1/SvImJ mice, which were specifically chosen because unlike C57BL/6J mice, this strain is not susceptible to hyperinsulinemia resulting from chronic glucocorticoid exposure, which could feedback and prevent increases in f(GNG) (22, 23, 24). We find, using the ^2^H_2_O labeling method, that dexamethasone treatment increases f(GNG) (0.553 ± 0.14 *versus* 0.230 ± 0.041, *p* = 0.0472) in ad-libitum fed mice (Fig. 3*C*). Additionally, we find that dexamethasone does not preferentially act on glycerol- or PEPCK-GNG (*i.e.* does not significantly alter the contribution of glycerol to the triose phosphate pool) in 24 h fasted mice (0.37 ± 0.17 *versus* 0.47 ± 0.21, *p* = 0.312) (Fig. 3*D*). More generally, this experiment is a case in point that the ^2^H_2_O labeling method can be used to establish or validate phenotypes in *in vivo* models.

### Confirmation of immunity to dilution from dietary glucose

MIDA uses ratios among enrichments instead of singular enrichments because ratios of enrichments are immune to dilution by natural abundance molecules (25, 26). In other words, the contribution of dietary glucose to blood sugar does not affect the calculated ratio of f(GNG) to f(GGL), because the ratios among enrichments of mass isotopomers are conserved. We validated this experimentally in context of this method by fasting mice for 24 h and briefly allowing them to refeed for 1 hour before sacrificing and drawing blood, with the expectation that this is not enough time to deposit and recycle glycogen and therefore obtain any signal from glycogen.

We find that there was no significant difference in *n* between both conditions (n = 5.74 *versus* 5.79, *p* = 0.775), which can be used to calculate f(GNG) (Fig. 4*B*). The similarity between these conditions implies that there was minimal flux into blood glucose from liver glycogen in this short time frame and, moreover, that fluctuations in dietary glucose levels are not likely to affect the f(GNG) results.

### Variation of glycerol contribution to triose-P and effect on f(GNG)

**Figure 5:**
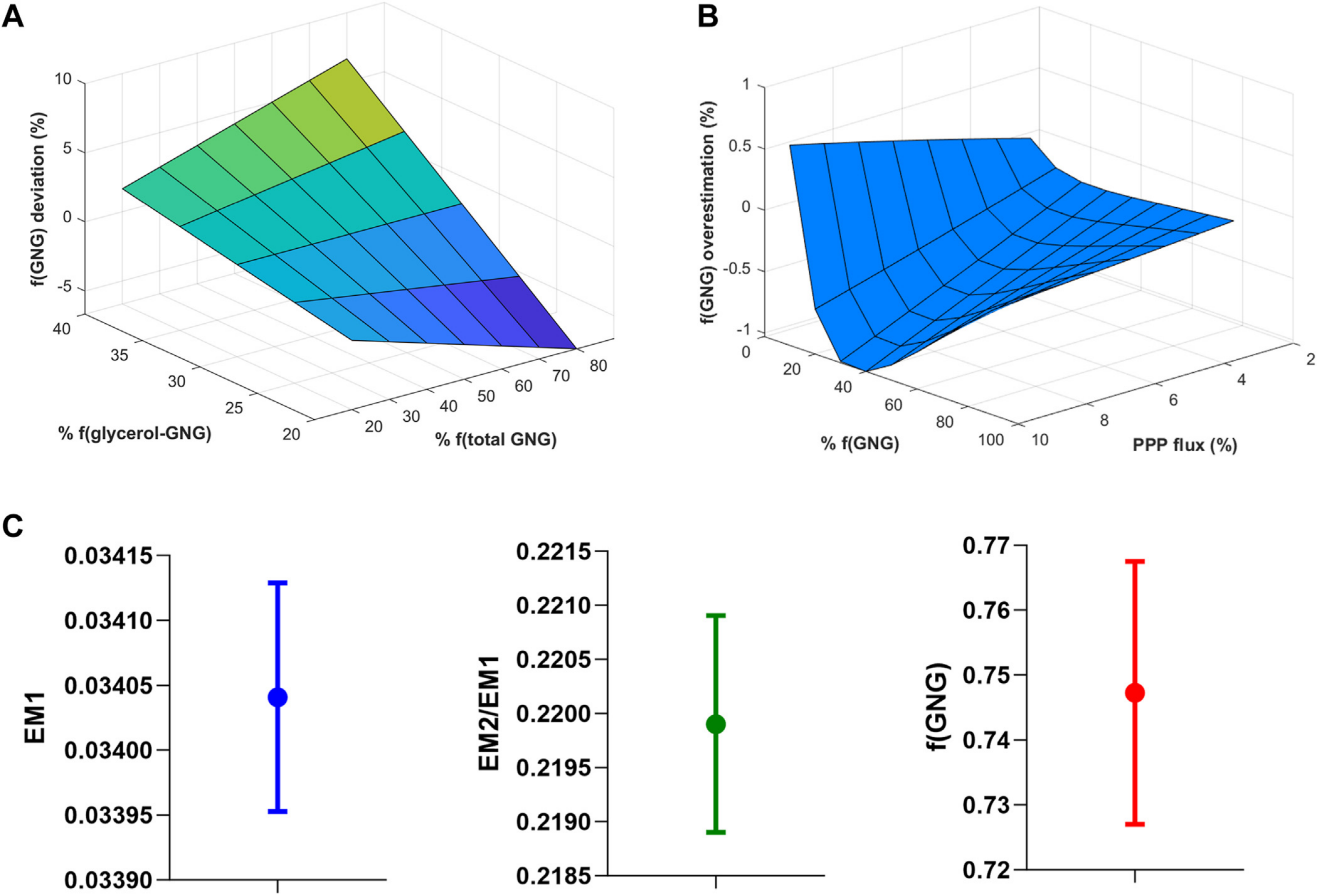
**Effects of factors potentially altering calculation of f(GNG) from heavy water.** *A*, simulation of variation in glycerol contribution to triose-phosphate pool and its effect on f(GNG) calculation using this model. Deviation in f(GNG) is shown on the vertical axis; (*B*), effect of transaldolase exchange on calculation of f(GNG). When f(GNG) >10%, transaldolase exchange introduces maximum 1% error; (*C*) technical replicate injections of a low enrichment sample and calculation of EM1, EM2/EM1, and f(GNG). The standard error in f(GNG) is 2%. GNG, gluconeogenesis.

Based on our results shown above and previously published literature, we make the simplifying assumption that glycerol carbons contribute roughly one-third of the triose-P pool (12, 14), such that *n* (total GNG) ≈ 6. Glycerol flux into triose-P could vary or fluctuate, however, as a function of metabolic or disease state or drug effect (16, 18). To address the potential quantitative impact of such variation, we examined the effect of fluctuation in glycerol contribution on the ultimate calculation of f(GNG). We performed a simulation where f(glycerol → TP) ranges 10% above and below the one-third assumption to encompass the spectrum of ranges reported in literature, and also varied f(GNG) from 20% to 80%, where f(glycerol → TP) is fixed at 33.3% (*i.e.* n = 6 for GNG pathway). The f(GNG) calculation with the variable f(glycerol-GNG) deviating from the assumption where n(GNG) = 6 resulted only a maximum of 6.7% error in the calculation of f(GNG), and that value only in the extreme case where f(GNG) is 80%. In most cases where f(GNG) is below 60% in a non-fasted state, the error is less than 4% (Fig. 5*A*). These results show that the error introduced by the primary assumption of this model is, in most cases, negligible or modest and of similar magnitude to inherent error in the general analytic technique.

### Negligibility of transaldolase exchange

One of the primary assumptions in many isotopic methods that measure GNG is the effect of transaldolase (TA) exchange (27, 28). During TA exchange, some of glucose-6-phosphate gets shunted into the nonoxidative pentose phosphate pathway (PPP), wherein redeposition of carbons occurs at the triose-phosphates. This would confer label on carbon-5 of glucose in the absence of true net GNG. This is a fundamental problem because label on carbon-5 in a positional isotopomer analysis is the only true proxy that encompasses total-GNG and not just PEPCK-GNG (10). Previous NMR studies have shown nonuniform labeling of carbons 3 and 4 in glucose when administered [U-^13^C]-glycerol and [1-^13^C]-acetate (27, 29, 30). The ^2^H_2_O labeling method described here quantifies the deuterium label distributed across all glucose C-H positions, not just C-5, so diversion of flux through pentose phosphate, where an extra label gets added to glucose position C-5, is expected in principle to have minimal effect on our results.

We performed a simulation whereby we added an extra label to glucose proportionally with PPP flux and calculate f(GNG) and compared it to f(GNG) calculated assuming no TA exchange. Only in the most extreme cases where f(GNG) is zero did this have any noticeable effect (Fig. 3*B*). When f(GNG) is greater than 10%, TA exchange introduces a maximum of 1.03% error.

### Accuracy of measurements with low body water enrichments

We performed the above experiments in mice, where it is possible to obtain body water enrichments of 4 to 5%. Higher body water enrichments result in higher enrichments of mass isotopomers (EM_x_). In the context of mass spectrometry measurements, this is commensurate with a higher signal-to-noise ratio.

This method is also intended for applications in humans in a clinical setting, however, which will typically involve a lower range for body water enrichments (*p*). Toward that end, we investigated analytical accuracy and effectiveness of this method at lower body water enrichments, around 1.0 to 1.5%, that are reflective of human studies using MIDA (31, 32, 33).

We injected technical replicates of the same sample into the GC-MS system and calculated EM1 (the enrichment of the M + 1 mass isotopomer at m/z 361), EM2/EM1, and ultimately f(GNG) to examine the variability in instrument output and its effect on final results. EM1 has a standard error of 8.88E-5 with a CV of 0.63%. EM2/EM1 has a standard error of 0.001 and f(GNG) has a standard error of 0.020 (2%).

These results suggest that the injection of technical replicates allows for accurate measurements of f(GNG) in human cohorts, whereby analytical error introduces a maximum of 2% standard error in f(GNG), setting the precedent for further studies in a clinical setting.

## Discussion

We present a method that calculates fractional GNG by mass spectrometric measurements of mass isotopomer enrichment patterns on blood glucose after metabolic labeling with heavy water. After ingestion of ^2^H_2_O, deuterium label gets incorporated into intermediary metabolites in the glycolytic, *de novo* amino acid synthesis, tricarboxylic acid cycle, and other pathways during enzymatic reactions where there is hydrogen-exchange with solvent water. Depending on the degree of and subpathway of fractional GNG, blood glucose will incur a differential amount and pattern of deuterium label, which is calculated by combinatorial analysis (15, 25).

Fractional GNG is defined as the fraction of glucose-6-phosphate-derived glucose that comes from the GNG pathway. The isotopic signature of glucose production pathways resulting from heavy water labeling is conserved when diluted with dietary glucose in the postabsorptive state (Fig. 4*B*). The constancy of isotopic ratios in the face of unlabeled dilution is a feature of MIDA which has been proven both mathematically (25) and here again experimentally, rendering this method applicable over different metabolic states of the study organism.

Rognstad *et al.* (34) showed that glucose C-2 is highly labeled from tritiated water because glucose-6-phosphate in liver rapidly equilibrates across the phosphoglucose isomerase reaction, where solvent hydrogen exchanges with C-2 of glucose during rapid isomerization (35). Rognstad *et al.* (11), also showed that in the presence of tritiated water, all seven nonacidic hydrogens in glucose become labeled from PEPCK-GNG so that *n* for PEPCK-GNG is seven. The more proximate and “true” precursor pool for GNG is the triose-phosphates, because that is where glycerol-GNG and PEPCK-GNG intersect (36). GNG measured using ^13^C-glycerol, which enters at the triose-phosphate pool and condenses into glucose, allows the use of combinatorial analysis or MIDA to calculate f(GNG) and the proportional contribution to triose-phosphate pool from free glycerol. We have shown previously (15, 37), and confirm here that free glycerol contributes ∼one-third to the triose-phosphate pool in both fasted and fed states. The present study is a variation on the MIDA approach using heavy water labeling instead, which has advantages over carbon-13 substrate labeling approaches.

Compared to other stable-isotope methods that measure GNG, heavy water is less expensive, does not require intravenous infusions, can be carried out on an outpatient basis and can be administered easily for longer periods of time. In addition, metabolic flux analysis with ^13^C tracers is susceptible to nonlinear propagation of error due to recycling of metabolites, while water distributes widely and almost immediately without compartmental differences and in theory can measure other metabolic fluxes in a single study (38). The previous MIDA method that we described for measuring GNG was with [2-^13^C_1_]-glycerol. Many other methods have been presented that use ^13^C-labeled substrates (39, 40, 41). The ^13^C-glycerol MIDA method is straightforward conceptually in that the substrate directly enters the precursor pool and, like most ^13^C-tracer studies, it involves an infusion rather than the simple oral consumption of water. A critique of the ^13^C MIDA method has been that metabolic zonation in the liver creates variational and disparate precursor enrichments (*i.e.* different *p* across the liver) (7, 42). This concern has been shown to have a quantitatively small effect even with extreme gradients across the liver (26), but heavy water labeling avoids any concerns about zonation across the liver. Other ^13^C methods that label at gluconeogenic substrates but do not estimate the true precursor pool to due dilution in the tricarboxylic acid cycle are not accurate quantitatively (43).

There are two commonly accepted methods for measuring GNG with heavy water: the C5-HMT method developed by (10) and the “average” method developed by Chacko *et al.* in 2008 (10, 44). The drawback of Landau’s method is that it involves significant labor at the bench to perform complex chemical reactions required to isolate carbon 5 on glucose. C-5 labeling from the nonoxidative portion of the pentose-phosphate cycle will also lead to overestimation of the contribution from net GNG (45). The “average” method circumvents chemical isolation of C-5 by analyzing fragments of glucose by GC-MS resulting from positive chemical ionization, and demonstrates that the inclusion of all carbons other than 2 is (approximately) equally effective at measuring GNG than the isolation of carbon 5 with the C5-HMT method (44). In addition, the only way that these other methods can be immune to dilution by dietary glucose (and calculate the fraction of EGP that comes from GNG) is by comparison to enrichment of carbon 2 to obtain a ratio, which requires extra labor and introduces more opportunity for analytical and/or experimental error. Using MIDA with heavy water labeling, as described here, is designed not to be affected by dilution from unlabeled glucose (Fig. 4*B*).

While GC-MS is a generally reproducible and robust platform to conduct measurements, reproducibility in mass fragmentation patterns of glucose to deconvolve the enrichment of carbon 2 in all GC-MS systems is sensitive to the various analytic parameters of the GC-MS system. Chemical ionization, which is used both in this method and the average method of Chacko *et al.* (44), uses low collision energies to avoid fragmentation of the analyte so that the parent ion, or the heaviest mass fragment is most abundant (46). On the other hand, electron ionization fragments the molecule much such that intact glucose may not even be detectable and offers less overall sensitivity (47). Analytical studies have examined the reproducibility of mass fragmentation patterns in GC-MS with chemical ionization and found that lower mass fragments are not as reproducible as the appearance of the parent ion (*i.e.* most intact and heaviest) because of the various parameters affecting ionization (48). Relying on mathematics (combinatorics, on which MIDA is based) provides a robust approach using the parent ion.

The major assumption of this method is setting the relative contribution of plasma glycerol carbons to the triose-phosphate pool, which we show here experimentally to be around one-third. Fixing the glycerol contribution reduces the problem such that fractional contributions from GNG and GGL are calculable, because this is otherwise an underdetermined system where a singular ratio EM2/EM1 does not contain enough information to inform on all three relevant pathway contributions (PEPCK-GNG, glycerol-GNG, and GGL), each of which has a unique *n*. Importantly, we show here that any physiologically reasonable discrepancy between the assumption and a particular individual introduces minimal error in the calculation of f(GNG). The average method (44) works on the same assumption, although it does not articulate this explicitly.

An assumption of most isotopic methods that measure GNG is the dismissal of TA exchange. NMR studies with ^13^C-labeled gluconeogenic substrates shows unequal labeling of carbons in glucose, demonstrating the significance of TA exchange and the potential problem with this assumption (27). Magnusson *et al.* (49) showed in 1988 that up to 6% of glucose could go into the pentose phosphate pathway in the liver. This value could be much higher depending on the mitotic state of the hepatocyte (50, 51). Glucose carbons, due to TA exchange, end up at the triose-phosphates and become labeled at C-5 in the absence of net GNG. The overestimation of f(GNG) using the C5-HMT method would therefore be directly proportional to PPP flux. In such a situation with heavy water labeling, the probability of obtaining one label on carbon-5, as dictated by a binomial, is 2∗*p*∗(1-*p*) which is always greater than *p* (except when *p* = 0.5) and is ∼2∗p at biologically feasible enrichments. Accordingly, GNG will be quantitatively overestimated. The method described here, in contrast, examines all carbons and is roughly an order of magnitude less sensitive to overestimation by TA-exchange than the C5-HMT method. Previous studies find that under the same conditions, f(GNG) measured with the C5-HMT method are higher than with [2-^13^C]-glycerol, in large part likely due to pentose phosphate cycling (7).

The ultimate aim of the current method for *in vivo* studies is application in humans in a clinical setting. The only difference with the results shown here, from an analytical standpoint, is that mice can be labeled to higher body water enrichments, which increases the signal to noise ratio of the enrichments of mass isotopomers (*e.g.* EM_x_), most notably that of EM2. At higher *p*, the difference between EM2/EM1 for GNG and GGL is also higher, proportionally increasing assay sensitivity. To address this concern, we injected technical replicates of mice with body water enrichments feasible in human studies (∼1–1.5%) (52, 53) and saw that accurate results are obtainable as well under these conditions, setting the precedent for human studies. One drawback of this method is that when f(GNG) is high, the analytical sensitivity at low enrichments becomes more difficult to resolve (Fig. 4*D*) because the curve has a high slope, which can be mitigated by multiple technical replicate injections.

In the compendium of methods that are used to measure the elusive GNG pathway, we introduce a new instance that builds on the trend of improvement with regards to accuracy and ease-of-use. This method leverages the convenience of heavy water administration and the robustness of combinatorial analyses to overcome many of the limitations found with other methods. Endogenous glucose production, of which GNG is a critical part, influences levels of one of the most important intracellular metabolites and plasma substrates, rendering its measurement of great importance in metabolic research.

## Experimental procedures

### Animals

Animal experiments were conducted according to the animal use protocol, approved by the UC Berkeley Animal Care and Use Committee. C57BL/6J male mice aged 8–12 weeks were acquired from the Jackson laboratory in Bar Harbor. Prior to specifying any experimental perturbation, mice were fed standard chow diet and water ad libitum. Mice were fasted for 24 h by the removal of CD from their cage. The mice that were refed were fasted for 24 h and then given moist CD inside the cage rather than inside the feeder to ensure a fast refeed for 48 min prior to sacrifice by anesthesia under isoflurane. Approximately, 400 μl of blood was drawn using a cardiac puncture. After the blood draw, mice were sacrificed and livers were also taken. Blood is kept on ice until being spun down for 8 min at 3200 rpm to separate plasma and erythrocytes, then stored at −80 degrees. Ad-libitum fed mice were labeled and sacked at the beginning of their dark cycle (6 PM to 10 PM).

### Heavy water labeling

Four hours prior to sacrifice, mice were given a bolus intraperitoneal injection of 100% D_2_O (Sigma-Aldrich 151882-250G) at 35 μl/g body weight, and then switched out the ad-lib drinking water at 8% D_2_O after the IP injection. For the lower enrichment experiments that mimic human conditions, the mice were instead given an IP of 100% D_2_O at 16 μl/g body weight and then provided with 2% D_2_O drinking water.

### 3-MPA administration

Mice were treated with 3-MPA (MedChem Express 320386-54-7) at 0.064 mg/g mouse. Administration of 3-MPA was done at the same time as heavy water labeling mixed in the 100% D2O.

### Glucocorticoid administration

Female 129S1/SvImJ mice aged 6 weeks ordered from Jackson Laboratories in Bar Harbor, were treated for 8 days hours with 2.4 mg/kg of dexamethasone (Supelco PHR1768) diluted in their drinking water. Mice were labeled similarly with D_2_O 4 h prior to sacrifice during their dark cycle with labeling beginning at 6 PM and sacrifice at 10 PM.

### [^13^C_1_]-glycerol infusions

C57BL/6J male mice 8 weeks old with jugular vein catheters inserted surgically were ordered from Charles River Laboratories. [2-^13^C_1_]-glycerol was purchased from Cambridge Isotope Laboratories (CLM-1397-0.25). Equipment for the infusion, which included a tether kit, the button attached to the catheter, and lever to hold the wire in place were supplied by Instech Inc. The infusate was a dilution of 36 μl of ^13^C-glycerol in 920 μl of 0.9% saline to achieve a concentration of 530 mM. After a bolus injection of 100 μl, the infusion was performed using a Harvard Apparatus 11 Plus Syringe Infusion Pump (#70-2208) at a flow rate of 0.16 μl/min/g for 3 h. Blood was collected immediately after the infusion using the protocol in the Animals section. Alternatively, blood was drawn from another catheter to avoid the viscous glycerol in the catheter causing an overestimate of blood glycerol enrichment.

### Glycogen content measurement

Glycogen Assay Kit from Sigma-Aldrich (MAK016-1KT) was used. Each sample, including standards, was loaded in duplicate. 10 μg of tissue were homogenized in 100 μl of water. A colorimetric assay on a 96-well plate was performed at 570 nm wavelength and corrected for background signal. A bicinchoninic acid assay was used to calibrate tissue weight as a function of protein mass. More detailed instructions are found in the manual of the referenced assay kit.

### Glucose extraction and derivatization

In a 1.5 ml Eppendorf tube, we aliquot 64 μl of plasma and then add 1 ml of an ice cold solution of 80:20 methanol:water. Each sample was vortexed for 20 s, then stored in −20 degrees for 10 min. The samples were then spun down at 16,000 rpm for 10 min. The supernatant was transferred to a GC-MS vial (*i.e.* to be placed in the autosampler) and dried down in a speedvac system.

In the same autosampler vial containing dried sample, we added 16 μl of acetonitrile to the dried sample to help it dissolve. We then added 72 μl of a freshly prepared mixture of excess methoxyamine hydrochloride (Sigma-Aldrich 226904-25G) in pyridine, and incubated the samples at 100 °C for 30 min (54). Then, we added 36 μl of acetic anhydride and incubate at 60 °C for 1 h, which produces the pentaacetate derivative. The mixture of organic solvents was dried under pressurized nitrogen and 800 μl of ethyl acetate was added as the solvent for the GC-MS analysis. It should be noted that this derivatization works by the aldehyde group reacting with methoxyamine and the hydroxyl groups with acetic anhydride.

### Mass spectrometric measurements

The system used is a Hewlett Packard (Agilent) 5973 quadrupole mass analyzer system coupled to an Agilent 6890N gas chromatography system with Agilent’s proprietary ChemStation software (https://www.agilent.com/en/product/software-informatics/analytical-software-suite/chromatography-data-systems/openlab-chemstation) used for data viewing and integration. The method was run on positive chemical ionization mode, and scanned for ions at m/z 360, 361, 362 as the M + 0, M + 1, and M + 2 mass isotopomers, respectively. This represents the methoxyaminated glucose penta-acetate derivative, but with one acetate group fragmented at a chemical formula of C_15_H_22_N_1_O_9_. The column used was a DB-225 (Agilent part #122 2962) with a retention time integration window of ± 0.04 min which only results in a single peak. If a DB-17 column (part #122 4732) is used, however, two peaks may be observed (55). This may represent the alpha and beta anomers of glucose or the E, Z stereoisomers resulting from different configurations of the aldehyde-bound methoxy group (56).

Careful attention must be paid to the concentration effect (57) because the labeling pattern and thus the enrichment of each glucose mass isotopomer can differ as a function of concentration of ions injected into the mass spectrometer source. Therefore, it is necessary to inject unlabeled standard prepared by the same derivatization protocol at varying concentrations to see which M + 0 abundance generates the closest to theoretical natural abundance for the M + 0, M + 1 and M + 2 isotopomers. This will determine what optimal amount of sample (*i.e.* at what M + 0 abundance) to inject for each sample analyzed.

### Body water enrichment analysis

Body water enrichment was calculated using the acetone method as described in (58). Briefly, 150 μl of the remainder of the blood drawn that is not used for glucose extraction (either plasma or erythrocytes) is distilled to recover the water. A total of 120 μl of the water is then set to incubate overnight in 2 μl of 10M NaOH and 4 μl of acetone. Then, adding 300 μl of hexane and vortexing will precipitate the acetone into the organic (hexane) phase. Two hundred microliters of the organic phase was inserted into a GC vial for injection into the GC-MS.

For measuring acetone, the mass spectrometer described in the previous section was set to electron ionization mode with an Agilent DB-225 column (part #122-2962) and scanned for ions at m/z 57, 58, and 59 for the M + 0, M + 1, M + 2 mass isotopomers, respectively.

Regression on a standard curve using water enriched at different levels will show the relationship between fractional M + 1 abundance and *p*, which will then calculate *p* for the sample of unknown enrichment. The sum of squared residuals (1-R^2^) for the standard curve, in our experiments, is below 0.003 (data not shown).

### MIDA calculations

There are three parameters in MIDA calculations: *n*, the maximum number of sites where there is hydrogen exchange with water and can therefore incur label, *p*, the enrichment of the precursor pool (*i.e.* isotopic enrichment of deuterated water), and *f*, the fraction of newly synthesized molecules.

For any given parameterization, there is a unique labeling pattern, which is defined as the enrichments (fractional abundances after subtraction of natural abundances) of the measured mass isotopomers. At any given *p* and *n*, the ratios among the enrichments (which in this study we measure as EM2/EM1) are conserved because enrichments scale linearly with *f* (25, 26).

Total GNG is a mixture of PEPCK-GNG which is characterized by incorporation of 7 H-atoms into C-H bonds of glucose (n = 7) and glycerol-GNG (n = 2). We generated experimental results (described below) to establish relative contributions from PEPCK-GNG and glycerol-GNG. We then mixed populations in these proportions in silico to obtain a labeling pattern for mixed GNG. We then mixed the labeling pattern of GNG with that of GGL (n = 1) at varying proportions of f(GNG), and calculated EM2/EM1. As a result, we can infer f(GNG) as a function of EM2/EM1, and generated at different isotopic enrichments *p* for reference in Table 1 using the following relationship:

$$\frac{EM2}{EM1}=\frac{\left(f\left(GNG\right)*EM2{|}_{n=6}\right)-\left(\left(1-f\left(GNG\right)\right)*EM2{|}_{n=1}\right)}{\left(f\left(GNG\right)*EM1{|}_{n=6}\right)-\left(\left(1-f\left(GNG\right)\right)*EM1{|}_{n=1}\right)}$$

Chen *et al.* (38) previously used a linear model to calculate flux ratios by calculating *n* instead of directly using R to obtain the flux ratio. Every *n* has a unique R (EM2/EM1) at a given *p* when *f* is 100%. The linear approximation (38) is accurate when pathway contributions do not have vastly different *n* or in situations where a small portion of flux ratio spectrum is under scrutiny, but becomes curvilinear at more disparate *n* (*e.g.* 1 *versus* 6 in this study (Fig. 4*D*), necessitating a mixture model as described in previous sections. Creating a line whereby EM2/EM1 is the independent variable and *n* is the dependent variable in direct proportionality allows for calculation of *n* from the experimentally derived EM2/EM1 by finding where it is located on the line (Fig. 4*D*). The calculated value of *n* is therefore a probability parameter delineating flux contributions from separate pathways each with a different *n*.

For this reason, *n* is an appropriate metric to quantify flux contributions in a tight window because it is not a function of *p* and resolves any variation in R resulting from different body water enrichments. While in theory any ratio R among mass isotopomers can be used (*i.e.* EM0/EM1), we find through experience that EM2/EM1 tends to be the most robust analytically for small molecules.

### Statistical analysis

Data between conditions were compared using an unpaired two-tailed Student’s *t* test, with a significance threshold (*i.e.* probability of accepting the null hypothesis H_0_) of < 0.05. Figures showing data from biological replicates and statistical analysis were performed using GraphPad Prism 9.0 (https://www.graphpad.com/features), with the error bars representing the SEM. Outliers were removed using the criterion of 1.5∗interquartile range.

### Simulations

MIDA tables to obtain labeling patterns for each pathway were generated using PyMIDA (https://github.com/naveedziari/PyMIDA). Simulations were conducted and corresponding figures were made in MATLAB R2019b. More detailed description of the simulations are provided in Results.

## Data availability

All data presented in the figures. Raw mass spectrometry data and code used for simulations and figures available upon request. Contact: naveedziari@berkeley.edu.

## Conflict of interest

The authors declare that they have no conflicts of interest with the contents of this article.
